# Morphological and Immunohistochemical Description of a Splenic Haemangioma in a Captive European Wolf (*Canis lupus lupus*) and a Review of the Current Literature

**DOI:** 10.3390/vetsci7030102

**Published:** 2020-08-03

**Authors:** Josep Maria Monné Rodríguez, Federico Morandi, Paolo Cavicchio, Alessandro Poli, Ranieri Verin

**Affiliations:** 1 Institute of Veterinary Pathology, Vetsuisse Faculty, University of Zurich, Winterthurerstrasse 268, CH-8057 Zürich, Switzerland; josep.monnerodriguez@uzh.ch; 2 National Park “Monti Sibillini” P.zza del Forno, 1 62039 Visso, Italy; federico.morandi@sibillini.net; 3 Pistoia Zoological Garden, Via Pieve a Celle Nuova, 160/A, 51100 Pistoia, Italy; paolocavicchio@tiscali.it; 4 Department of Veterinary Science, University of Pisa, 56124 Viale delle Piagge, 2 56124 Pisa, Italy; 5 Department of Comparative Biomedicine and Food Science—University of Padova AGRIPOLIS—Viale dell’ Università 16, 35020 Legnaro, Italy; ranieri.verin@unipd.it

**Keywords:** european wolf (*Canis lupus lupus*), haemangioma, histopathology, immunochistochemistry, spleen

## Abstract

Neoplastic diseases are rarely described in wild carnivores; only a few reports have been published on this topic. Here, we describe the histological and immunohistochemical features of a haemangioma in the spleen of a grey wolf (*Canis lupus lupus*) and we compare the results with the dog (*Canis lupus familiaris*). Additionally, we list the different publications found in the literature with neoplastic lesions in wolves. Our results show similar immunohistochemical features to dogs, in which neoplastic cells express Vimentin, von Willebrand factor, alpha smooth muscle actin antibody, vascular endothelial growth factor C and low vascular endothelial growth factor receptor 3. Toluidine blue special stain shows moderated increased numbers of mast cells infiltrating the tumor, a feature observed in benign vascular tumors in domestic dogs, but not in the malignant counterparts. To our knowledge, this is the first article describing the gross, histological and immunohistochemical features of a splenic haemangioma in a wolf.

## 1. Introduction

In contrast to studies on dogs, a limited number of reports describing tumors in wild carnivores are found in the literature. This is particularly noticeable in species belonging to the genus *Canis*, for which only a few reports usually referring to single cases or large surveys of populations without specific histopathological and immunohistochemical details are described.

Haemangioma and haemangiosarcoma are common in dogs (*Canis lupus familiaris*), the dermis and subcutis being the most common primary sites for the haemangioma, and the spleen, skin, subcutis, right atrium and liver being the most common sites for haemangiosarcoma [1]. In contrast to dogs, only one case of a splenic haemangioma is reported in wolves; this case refers to a 10-year-old captive red wolf (*Canis rufus*) with a concomitant thyroid carcinoma and two adrenal myelolipomas and no histological information is provided [2]. Therefore, this paper had the aim of describing, for the first time, the gross, histopathological and immunohistochemical characteristics of a splenic haemangioma in a captive European wolf (*Canis lupus lupus*) and comparing the results with previously published studies on dogs as well as providing a list of the different publications describing neoplastic lesions in wolves.

## 2. Materials and Methods

### 2.1. Subject

The present subject was a nine-year-old male captive European wolf (*Canis lupus lupus*) from a zoological garden that was presented for necropsy examination after it had been humanely euthanized due to chronic emaciation and apathy with no response to treatment.

### 2.2. Pathological Investigations

After a complete necropsy, representative tissue samples were fixed in 10%. A complete necropsy was performed and representative tissue samples from the main visceral organs, including liver, lungs, brain, spleen and splenic mass, were fixed in 10% neutral buffered formalin and routinely processed for paraffin embedding. Four-micron-thick sections were stained with Hematoxylin and Eosin, Goldner’s Trichrome and Periodic-Acid Schiff, Perls, and Toluidine Blue (TB) stains. For immunohistochemical investigations, four-µm-thick sections were de-waxed in xylene, hydrated throughout a graded series of ethanol, and rehydrated in deionized water. Antigen retrieval was performed with citrate buffer pH 6.0 in a microwave oven for 5 min at 750 W and 13 min at 350 W and cooled at room temperature. After exhausting endogenous peroxidases activity with Peroxidase blocking solution^®^ (Dako, Glostrup, Denmark), non-specific reactions were blocked by incubating each section with two drops of Ultra V-block^®^ (ThermoFisher Scientific; Waltham, MA, USA). Primary antibodies were incubated overnight in a moist chamber. The primary antibodies used were a mouse monoclonal anti-vimentin antibody (clone V9, dilution 1:100; Dako M0725, Glostrup, Denmark), a rabbit polyclonal anti-vascular endothelial growth factor C antibody (VEGFC; Zymed laboratories Inc., San Francisco, CA, USA), a rabbit polyclonal anti-vascular endothelial growth factor receptor 3 antibody (VEGFR-3; Alpha Diagnostic International, San Antonio, CA, USA), a rabbit polyclonal anti-von Willebrand factor antibody (vWF/Factor VIII; Dako), a mouse monoclonal anti-alpha smooth muscle actin antibody (α-SMA; Dako), and a mouse monoclonal anti-CD44 antibody (Dako). After washes with Tris-buffered saline (TBS) plus and 0.1% Triton X-100 TBST, this phase was followed by incubation with a universal polyvalent biotinylated antibody (Horse anti mouse/rabbit IgG RTU, Vector Lab., Burlingame, CA, USA) for 15 min. After washing with TBST, streptavidin-peroxidase complex (Vector Lab. Burlingame, (CA) USA) was incubated for 15 min. Peroxidase activity was revealed by incubation for 10 min in 3,3′-diamonibenzidine tetrahydrochloride (ImmPACT DAB Peroxidase Substrate Kit^®^, Vector Labs inc., Burlingame, CA, USA) and blocked with deionized water. Finally, sections were counterstained with Mayer’s Haematoxylin, dried and covered with cover slips. Negative controls were performed by replacing the primary antibody with a unrelated mouse isotype matched control monoclonal antibody (clone MA5-14453; TermoFisher Scientific, Rockford, IL, USA) and a rabbit polyclonal anti-toxoplasma antibody. This immunohistochemical panel has been previously used in a comparative study of canine vascular tumors [3]. Tumors were classified according to the WHO classifications of tumors of domestic animals [4].

## 3. Results

**Figure 1:**
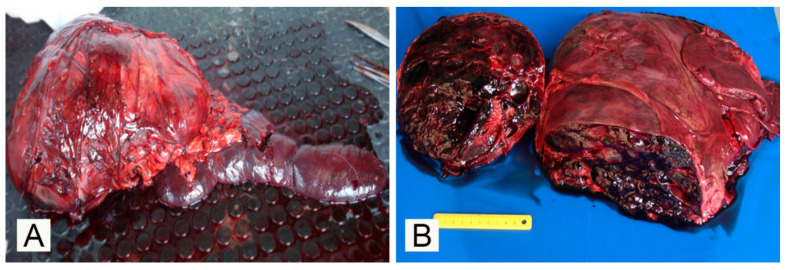
Gross presentation of the splenic mass in a nine-year-old male European wolf (*Canis lupus lupus*): (**A**) Gross presentation of the spleen with a focal round to oval mass bulging the splenic capsule; (**B**) On the cut surface, the splenic mass shows blood-filled cavernous spaces.

Upon macroscopic examination, the spleen exhibited a large, ~40 × 10 × 15 cm, nodular, poorly demarcated, mass with a similar consistency and color to the remainder of the spleen, growing from the ventral aspect of the organ and bulging on the surface of the organ (Figure 1A). On the cut surface, the mass showed numerous, variably sized, up to 1 cm in diameter, blood-filled spaces with a cavernous pattern (Figure 1B). The mass occupied the cranial half of the abdominal cavity and displaced the adjacent viscera. The remainder of the spleen appeared diffusely and severely congested and oozed abundant blood on cut surfaces.

**Figure 2:**
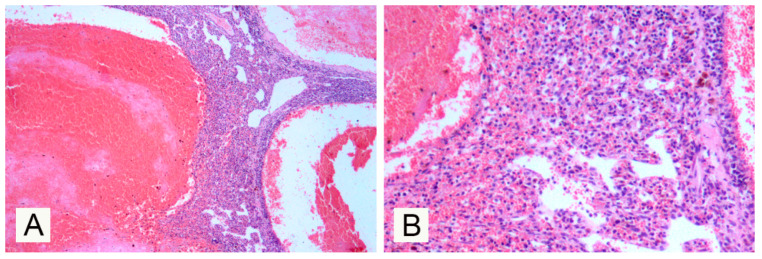
Histological photomicrograph of the splenic mass: (**A**) Cavernous pattern with large, regular and well-defined vascular spaces filled with erythrocytes (Haematoxylin-Eosin stain, Ob. 10×); (**B**) higher magnification, areas with a neoplastic capillary pattern (Haematoxylin-Eosin stain, Ob. 20×).

Histologically, the mass was composed of a poorly cellular, well demarcated, partially encapsulated and expansile neoplastic proliferation forming large blood-filled cavernous spaces (Figure 2A) separated by a thin fibrous stroma and lined by a single layer of spindled and flattened cells (neoplastic endothelial cells). Anisokaryosis and anisocytosis were mild, and no mitotic figures were counted in 10 high-power fields. Mixed with these large blood-filled cavernous spaces, more cellular areas were observed where neoplastic cells tended to give small neoplastic capillary structures (Figure 2B). At the periphery of the neoplastic lesion and in normal tissue entrapped within the neoplastic tissue there were scattered inflammatory cells, including macrophages, often with cytoplasmic brown granular pigment (hemosiderin; hemosiderophages) and a few lymphocytes and plasma cells. Inflammatory cells, mainly viable neutrophils, were occasionally observed in contact with the neoplastic endothelium lining neo-formed blood vessels.

**Figure 3:**
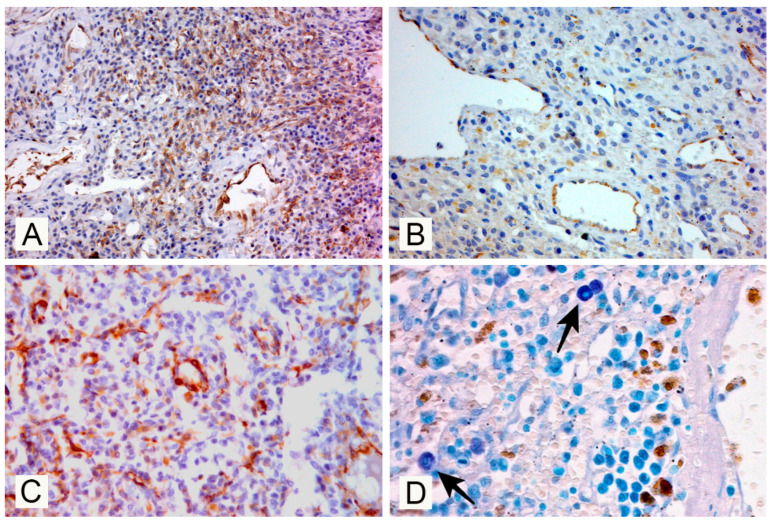
Immunohistochemical expression of the different markers used and the Toluidine Blue (TB) stain of the splenic neoplastic tissue: neoplastic cells show a positive signal for vimentin (**A**), von Willebrand factor, (**B**) and alpha smooth muscle actin (**C**) (Immunohistochemical staining, Haematoxylin counterstainig); (**D**) TB special stain showing a moderate number of infiltrating mast cells (arrows).

The results of immunohistochemical and histochemical investigations are presented in Figure 3.

As expected, neoplastic cells were strongly positive for vimentin (Figure 3A). The vascular origin of the neoplastic cells was confirmed by the diffuse expression of vWF protein, a marker for endothelial cells (Figure 3B) [5]. The strong α-SMA signal expressed by the neoplastic cells suggest an intrinsic contractile capacity of neoplastic endothelium (Figure 3C). Neoplastic endothelial cells as expected scored negative for CD44 antigen. A moderately increased number of mast cells (showing magenta staining with TB) was observed infiltrating throughout the neoplasm (Figure 3D) if compared to the adjacent not-affected splenic parenchyma. The other organs sampled for histopathology did not show evidence of micro-metastases, and the main change that was constantly observed was a chronic congestion mainly evident in the splenic parenchyma adjacent to the mass.

## 4. Discussion

Neoplastic lesions are rarely described in wolves and little is known about the incidence in this species. A list of the main publications about neoplastic diseases in wolves is made available in Table 1, and evidence of multiple tumors in the same animal is reported. As is evident from Table 1, there is a higher incidence of malignant epithelial tumor (carcinomas) if compared to mesenchymal and round cell tumors in the species listed below.

The main gross change that could explain the clinical signs reported and that led to euthanasia was the massive compression of venous return from the abdominal compartment due to a large expansive splenic mass impeding the normal blood flow in the caudal vena cava. Since there is no data in literature describing histological features of vascular tumors in wolves, our results provide further insights on splenic hemangioma in this species. Vascular tumors are common in dogs and may arise in any vascularized tissue [13,14] and the most affected organs in this species are the spleen, right atrium and liver [15,16]. Canine hemangiosarcoma are locally infiltrative and readily metastasized, particularly to the lung and liver [17] and despite surgical or chemotherapeutic management, affected animals have a poor prognosis with a survival time little more than six months [18,19,20]. The main differential diagnosis for a large splenic mass like the one observed in the present case, excluding hemangioma, is splenic hematoma and hemangiosarcoma, although the latter seems less likely considering such a large mass not associated with clinical signs or evidence of gross and micro-metastases in the main organs sampled. Splenic hematoma and hemangiosarcoma are far more common than hemangioma in dogs (*Canis lupus familiaris*) [4]. This is not known in wolves; however, even though hemangiosarcoma has never been described in this species, the lack of publications makes difficult to know with certainty the real incidence. Nevertheless, our results show that the immunohistochemical features of the current hemangioma have clear similarities with the features described in dogs, in which VEGFR-3 was less expressed in benign vascular tumors (as in this case) and neoplastic cells show a positive signal for Vimentin, vWF, α-SMA and VEGFC. VEGFC and vascular endothelial growth factor D are both vascular endothelial growth factors (VEGFs) involved in the maintenance of normal adult endothelial cells, proliferation and the increase in vascular permeability and were found expressed by human vascular neoplastic cells [5,21,22,23,24,25]. In a previous study on canine vascular tumors, VEGFR-3 expression was found in the majority of hemagiosarcomas investigated, suggesting that this marker would not be suitable for the immunohistochemical discrimination between malignant and benign vascular tumors [26,27,28]. In contrast, the expression of VEGFC has been found limited to neoplastic endothelial cells in hemangiomas with capillary differentiation [3]. In the current case, neoplastic cells showed moderate intracytoplasmic positivity for VEGFC and a mild positive signal for VEGFR-3. CD44 protein is an adhesion molecule that plays a role in cell migration and is overexpressed in different malignant tumors [29]. In our study, endothelial cells of the benign vascular tumor detected in the wolf were also CD44-negative, confirming a previous study on canine vascular tumors where all the hemangiomas investigated failed to express this marker, whilst more than half of the malignant vascular tumors investigated were immunoreactivity for this marker [4]. CD44 can be also expressed by the proliferating endothelial cells of granulation tissues, confirming their molecular similarity with malignant ones [3], but gross and histopathological aspects of the lesion described here allowed to exclude a reactive vascular proliferation. A specific immunohistochemical study for lymphatic endothelial markers (to rule out a neoplasm of lymphatic origin) was not performed, as moderately differentiated blood-filled channels, morphologically consistent with neoplastic blood vessels, reinforce the diagnosis of haemangioma. In dogs, the number of infiltrating mast cells was found to be different between malignant and benign vascular tumors; mast cells were numerous in the majority of hemangiomas but less commonly found in granulation tissue and hemangiosarcoma [3]. Interestingly, our findings show that there are indeed mast cells in moderate numbers infiltrating the benign neoplastic process, and this is also consistent with previous findings in dogs [3].

## 5. Conclusions

To our knowledge, the current report describes, for the first time, gross, histological and immunohistochemical features of a splenic haemangioma in a wolf. Although this finding could suggest some differences between species, the reduced available information in literature and the single subject examined here limit the interpretation of our results in this species.

## Figures and Tables

**Table 1:** List of current publications describing neoplasm in wolves including species and main pathological findings.

Species	Main Findings	Reference
Mexican gray wolf (*Canis lupus baileyi*)	Malignant mammary tumor	[6]
European wolf (*Canis lupus lupus*)	Subcutaneous leiomyosarcoma	[7]
Polar wolf (*Canis lupus arctos*)	Oral squamous cell carcinoma	[8]
Red wolves (*Canis rufus*)	Two multicentric lymphomas, one mesenteric round cell tumor, one bronchial carcinoma and two osteosarcomas	[2]
American coyotes (*Canis latrans*) and wolves (*Canis lupus*).	Oral papillomatosis	[9]
Red wolves (*Canis rufus*)	Forty-three cases in which carcinoma or adenocarcinoma and lymphoma were the most common. There were also reports of pheochromocytomas, osteo-sarcomas, fibrosarcomas, granulosa cell tumors, sarcomas, lymphosarcomas, nerve sheath tumors, sertoli cell tumors, histiocytomas, and adenomas.	[10]
Eurasian wolf (*Canis lupus lupus)*	Pulmonary neuroendocrine tumor	[11]
Gray wolf (*Canis lupus*)	Papillomaviral plaque	[12]
